# The risk of antidepressant-induced hyponatremia: A meta-analysis of antidepressant classes and compounds

**DOI:** 10.1192/j.eurpsy.2024.237

**Published:** 2024-08-27

**Authors:** T. Gheysens, L. De Picker, F. Van Den Eede

**Affiliations:** ^1^Psychiatry, AZ Klina, Brasschaat; ^2^SINAPS, Duffel; ^3^CAPRI, Antwerp; ^4^Psychiatry, Antwerp University Hospital, Edegem, Belgium

## Abstract

**Introduction:**

Hyponatremia (hypoNa) is a potentially serious adverse event of treatment with antidepressants. Previous research suggests that risk of drug-induced hyponatremia differs between antidepressants.

**Objectives:**

This meta-analysis sought to determine the risk of antidepressant-induced hypoNa, stratified by different compounds and classes.

**Methods:**

PubMed and Web of Science were searched for studies reporting on incidence or risk of hypoNa in adults using antidepressants (PROSPERO, CRD42021269801). We modelled random-effects meta-analyses to compute overall incidence and risk of any and clinically relevant hypoNa for each compound and class, and ran head-to-head comparisons based on hypoNa incidences. We conducted subgroup analyses for geriatric populations, study context and sodium cut-off value.

**Results:**

Thirty-nine studies (n = 8,459,033) revealed that exposure to antidepressants was associated with significantly increased odds of hypoNa (OR = 2.82 (1.79 – 4.45)). The highest event rates were found for SNRIs (7.17%), SSRIs (5.20%), and TCAs (2.26%); the lowest for mirtazapine (1.02%) and trazodone (0.89%). The highest odds ratios were found for MAOIs (4.12 (1.92 – 8.86)), SNRIs (3.16 (1.77 – 5.67)), and SSRIs (2.78 (1.57 – 4.91)); the lowest for mirtazapine (2.82 (1.87 – 4.21)) and TCAs (1.85 (1.28 – 2.69)). Compared to SSRIs, SNRIs were significantly more likely (OR = 1.27 (1.13 – 1.42), p < 0.001) and mirtazapine significantly less likely (OR = 0.61 (0.39 – 0.96), p = 0.032) associated with hypoNa.

**Conclusions:**

Our meta-analysis demonstrated that, while no antidepressant can be considered completely risk-free, for hypoNa-prone patients mirtazapine should be considered the treatment of choice and SNRIs should be prescribed more cautiously than SSRIs and TCAs.

**Disclosure of Interest:**

None Declared

